# Cortical myoclonus and epilepsy in a family with a new *SLC20A2* mutation

**DOI:** 10.1007/s00415-020-09821-4

**Published:** 2020-04-09

**Authors:** Antonietta Coppola, Laura Hernandez-Hernandez, Simona Balestrini, S. Krithika, Nicholas Moran, Blake Hale, Carla Cordivari, Sanjay M. Sisodiya

**Affiliations:** 1 Epilepsy Centre, Department of Neuroscience, Reproductive and Odontostomatological Sciences, Federico II University, Naples, Italy; 2 Department of Clinical and Experimental Epilepsy, UCL Queen Square Institute of Neurology, Queen Square, London, WC1N 3BG UK; 3 The Chalfont Centre for Epilepsy, Chalfont-St-Peter, Bucks, UK; 4 East Kent Hospitals University Foundation Trust, Ethelbert Road, Canterbury, Kent UK; 5 Department of Clinical Neurophysiology, UCL Queen Square Institute of Neurology, London, UK

**Keywords:** *SLC20A2*, Brain calcifications, Epilepsy, Cortical myoclonus, Exome sequencing

## Abstract

Idiopathic basal ganglia calcification (IBGC) or primary familial brain calcification is a rare genetic condition characterized by an autosomal dominant inheritance pattern and the presence of bilateral calcifications in the basal ganglia, thalami, cerebellum and cerebral subcortical white matter. The syndrome is genetically and phenotypically heterogeneous. Causal mutations have been identified in four genes: *SLC20A2*, *PDGFRB*, *PDGFB* and *XPR1*. A variety of progressive neurological and psychiatric symptoms have been described, including cognitive impairment, movement disorders, bipolar disorder, chronic headaches and migraine, and epilepsy. Here we describe a family with a novel *SLC20A2* mutation mainly presenting with neurological symptoms including cortical myoclonus and epilepsy. While epilepsy, although rare, has been reported in patients with IBGC associated with *SLC20A2* mutations, cortical myoclonus seems to be a new manifestation.

## Introduction

Idiopathic basal ganglia calcification (IBGC) or primary familial brain calcification (PFBC) is a rare autosomal dominant genetic condition characterized by the hallmark of bilateral calcifications in the basal ganglia, thalami, cerebellum and subcortical white matter. The clinical presentation can be widely heterogeneous even among members of the same family. While the imaging phenotype shows 100% penetrance (all patients carrying mutation have calcifications by the age of 40–50 years), the clinical phenotype only shows 61% penetrance [11, 19, 24]. The clinical phenotype typically includes neurological and psychiatric features [11, 18, 19, 24].

The condition is also heterogeneous at the genetic level, mutations in four genes have been reported: *SLC20A2*, *PDGFRB*, *PDGFB* and *XPR1* [16, 19]. Mutations in *SLC20A2* seem to be the major cause of IBGC, accounting for as many as 41% of the familial cases [11]. This gene encodes the inorganic phosphate (Pi) transporter PiT2 which is expressed ubiquitously in the brain, mainly in the sites where calcifications tend to occur (the globus pallidus, thalamus and cerebellum) [6]. *Slc20a2* knockout mice show elevated cerebrospinal fluid (CSF) [Pi], suggesting that the sodium-dependent phosphate transporter-2 (PiT2) is essential for [Pi] export from the CSF, and thus regulation of CSF [Pi] [13].

Mutations or deletion in *SLC20A2* are associated with psychiatric conditions (56.9%) including bipolar disorder, mood disturbances and psychosis [2, 19] and neurological symptoms mainly affecting cognitive abilities (58.8%) and motor function (54.9%), including dystonia, chorea, parkinsonism and ataxia [19]. Migraine seems also to be common, but the specific prevalence is difficult to ascertain given the high prevalence in the general population [20]. Epilepsy has been reported more rarely (7.8%) [17, 19]. Given the co-occurrence of movement disorders, neurophysiology (video-polygraphy) is essential to classify epilepsy and differentiate seizures from abnormal movements [14].

Here we report on a family affected by IBGC, showing an autosomal dominant inheritance and a newly reported *SLC20A2* mutation. The clinical presentation of this family is complex and includes cortical myoclonus and epilepsy as the main features. While epilepsy has been reported in seven cases with IBGC and *SLC20A2* mutations [1, 8, 11, 14, 25, 30], cortical myoclonus has not been reported to our knowledge.

## Methods

The study was approved by the appropriate ethics committee. Written informed consent was provided by the family.

The proband was clinically evaluated through a comprehensive neurological and psychiatric examination at the National Hospital for Neurology and Neurosurgery, London. Brain Computed Tomography (CT) scan, electroencephalography (EEG), and peripheral nerve assessment were performed. Genomic DNA of the patient and her mother was extracted from peripheral blood. The father was not available for sampling.

An exonic library was amplified by Nextera Exome Kit (Illumina), and sequenced on a HiSeq 2500 platform (Illumina). Alignment of reads was carried out against the reference human genome (GRCh37). Candidate genes were analyzed by vcf tools [7] and annotated using Annovar [28]. Sanger sequencing was performed to confirm the variant.

## Family description

**Fig. 1:**
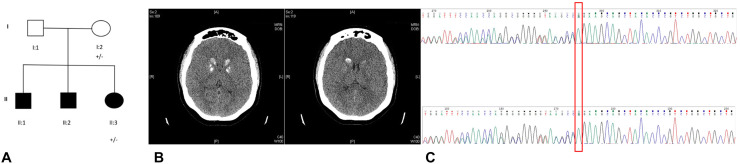
**a** pedigree of the family; filled symbols indicate subjects with symptoms; ± indicates screened individuals with heterozygous *SLC20A2* mutation; **b** CT scans of the proband showing calcification of the basal ganglia and caudate nuclei. Further diffuse calcification is noted within the region of the centrum semi-ovale on the right; **c** electropherogram from the proband (lower box) and her mother (upper box)

This is a two-generation family of British origin (Fig. 1a). The proband (II:3) is a 22-year old woman, the third child of unrelated parents. She was born at term through a natural delivery after an uneventful pregnancy. The psychomotor milestones were normal. At the age of eight she was noticed to have myoclonic jerks affecting her hands and legs, particularly in the morning. The interictal EEG at that time showed bursts of bilateral spike and slow wave activity. The jerks were resistant to topiramate and levetiracetam (LEV) treatment. A concomitant slowing in school performance was reported. From the age of 11 she experienced anxiety and phobias. At the age of 14, she underwent a comprehensive psychiatric evaluation for autism and intellectual disabilities: she was assessed using the Autism Diagnostic Observation Schedule-Generic (ADOS-G) and the Wechsler Intelligence Scale for Children, 4th edition (WISC-IV). The diagnostic criteria for Autism Spectrum Disorders were not met, although social communication and social interaction difficulties were present. Her IQ fell within the low average range. At the age of 14 she had her first GTCS and these recurred every few months. Around the same age, she also developed dystonic attacks involving her upper limbs and spreading to the neck and torso. They could last from 1 h up to 24 h. These involuntary movements worsened her mood disturbances and had a significant impact on her mobility, requiring her to use a wheelchair when not at home.

At the age of 18, she was first admitted to our hospital and underwent a comprehensive evaluation. Generalized tonic–clonic seizures (GTCSs) had ceased, but she had very frequent dystonic attacks. These affected all four limbs, her head, back, and abdomen. Sometimes these were triggered by environmental light and were always preceded by generalized jerks and a feeling of fear. On admission, she was on treatment with valproate (VPA) 1100 mg/day, LEV 1000 mg/day and clonazepam 3 mg/day.

On examination, she was able to walk unassisted but preferred to use a wheelchair outdoors. Her gait was slightly ataxic, without upper limb ataxia, but rare myoclonic upper limbs jerks were noted. Cogwheel rigidity was present in both arms.

Psychometry revealed a low average score on Verbal Comprehension (85), Perceptual Reasoning (84) and working memory (83) subscales and a borderline score in the processing speed (76) subscale of the WAIS-IV (Wechsler Adult Intelligence Scale). The full scale IQ score was 79.

CSF, including testing for autoantibodies associated with encephalitic presentations, was unremarkable. Blood and urine organic acids went normal. Serum calcium and parathyroid hormone levels were normal. A brain MRI showed bilateral symmetrical signal abnormality of the globus pallidus, head of caudate and nucleus accumbens, consistent with abnormal mineralization.

At the age of 21, a CT scan was performed, showing calcification of the basal ganglia and caudate nuclei. Further diffuse calcification was noted within the region of the centrum semi-ovale on the right (Fig. 1b).

The awake EEG showed brief high voltage spikes and slow waves. Several episodes characterized by four limb shaking with retained consciousness were captured, without any EEG correlate, suggesting these episodes were not epileptic.

**Fig. 2:**
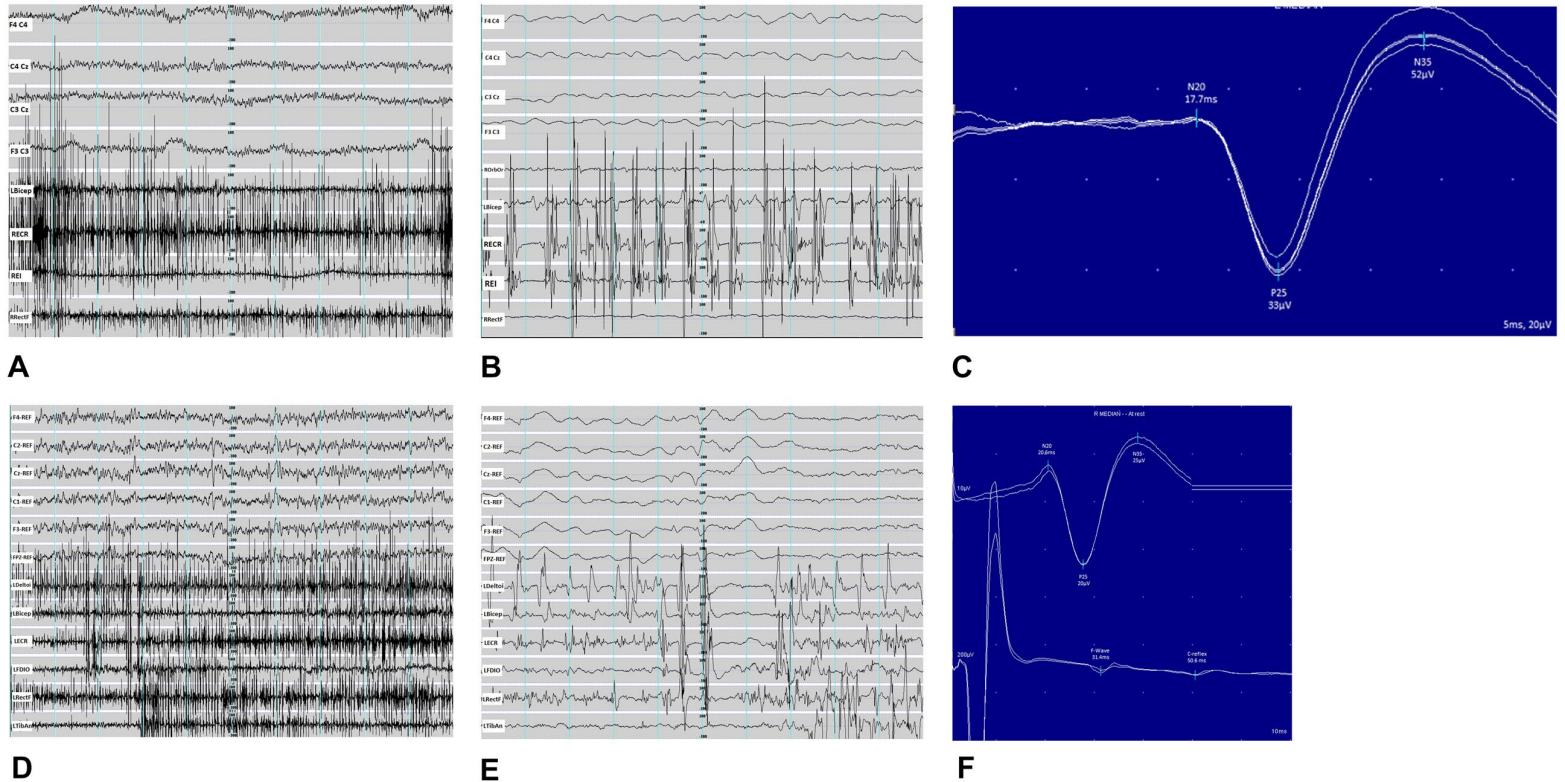
Neurophysiology study of the proband and her brother II:2. **a**, **b** EEG-EMG recording of the proband showing positive and negative myoclonic jerks when extending arms against gravity. **a** The trace (1.0 s/division) shows the ongoing 20 Hz myoclonic jerks; **b** the trace (0.1 s/division) demonstrates craniocaudal progression of atonic periods on surface EMG (bipolar EEG channels; EMG channels: *ROrbOr* right orbicularis oris, *LBicep* left bicep, *REI* right extensor indicis, *RECR* right extensor carpi radialis, *RRectF* right rectus femoris). **c** Somatosensory evoked potential of the proband, recorded from the median nerve showing a giant evoked potential (P25–N35 = 52 µV) at the scalp electrodes (C4-Fz). **d**, **e** EEG-EMG recording of the brother II:2 showing positive and negative myoclonic jerks when extending arms against gravity. **d** The trace (1.0 s/division) shows the ongoing 20 Hz myoclonic jerks; **e** the trace (0.1 s/division) demonstrates craniocaudal progression and atonic periods on surface EMG channels. [EEG channels (F4, FC4, Cz, FC3, F3, FPZ) referenced to linked earlobes]; EMG channels top–bottom: *LDeltoi* left deltoid, *LBicep* left bicep, *LECR* left extensor carpi radialis, *LFDIO* left first dorsal interosseous, *LRectF* left rectus femoris, *LTibAn* left tibialis anterior). **f** Evoked potential recording showing ‘giant’ (P25–N35 = 25 µV) response at the scalp electrodes (C3-Fz) and cortical-reflex (C-reflex, 50.6 ms) from the surface abductor pollicis brevis electrode

A multichannel EEG-EMG recording showed a pattern of frequent myoclonic jerks of short duration with craniocaudal progression and intermittent silence of muscle activity, suggestive of positive and negative cortical myoclonus (cortical tremor; Fig. 2a, b). A giant SEP was also found supporting the presence of cortical hyperexcitability (Fig. 2c).

The eldest brother (II:1) was born by natural delivery after an uneventful pregnancy; he is now 27 years old. He had normal developmental milestones. He was diagnosed with high-functioning social communication disorder (Asperger’s Syndrome) at the age of 12 years. He developed myoclonic jerks at the age of 11 years. At that time, EEG showed bilateral spike and polyspike and wave activity; these abnormalities increased on photic stimulation at flash frequencies between 15 and 20 Hz. He also reported blackouts, but absences were never clinically observed nor caught on EEG recording. During treatment with carbamazepine (CBZ) he had GTCSs. He was then switched to VPA (1600 mg/day) with a significant reduction of the jerks. However, because of weight gain, VPA had to be discontinued, and was substituted with LEV (up to 1500 mg/day). He underwent a brain MRI twice (13 years old and 22 years old): both exams were reportedly normal.

The second brother (II:2) is now 24 years old. He had normal developmental milestones. At the age of 10 years, he had his first nocturnal GTCS and from the age of 12 he noted small myoclonic jerks of his hands. He was initially treated with CBZ, which failed to control seizures and worsened the jerks. He was then switched to VPA; LEV was added as seizure control was not complete. EEG performed at the age of 11 showed bursts of high voltage poly-spike and slow wave discharges, more frequent during drowsiness and during intermittent photic stimulation. From the age of 17 he had daytime GTCS and myoclonic jerks worsened. Clonazepam (up to 2 mg/day) was added to VPA (1200 mg/day) and LEV (500 mg/day), but myoclonus has never been controlled. Propranolol (40 mg/day) was added at the age of 20. A brain MRI performed at the age of 18 was normal. A neurophysiology study was performed at the age of 23 including an EEG-EMG (Fig. 2d, e). No myoclonic jerks were seen at rest. When holding his arms against gravity and during action, 20 Hz myoclonic jerks lasting about 20 ms, and episodes of sudden loss of tone of about 100 ms, were recorded in all the EMG channels. These abnormal findings together with a positive C-reflex and giant SSEP, were consistent with cortical myoclonus (Fig. 2f).

The mother I:2 has never reported psychiatric nor neurological symptoms.

## Genetic results

A heterozygous mutation in *SLC20A2* was identified in two members (see Fig. 1a: proband II:3 and mother I:2). DNA was not available from father and brothers (Fig. 1a: II:1 and II:1, II:2, respectively).

The deletion (c.1583delA) located in exon 9 causes a frameshift (p.Q528fsD) (see Fig. 1c) predicting a truncated protein. This mutation is absent from public databases (ExAc, Exome Variant Server, 1000Genomes, gnomAD) and was predicted as damaging by SIFT. Polyphen-2 is only available for amino acid substitutions; it is not available for this mutation but the predicted CADD score is 35. Sanger sequencing confirmed the mutation in both individuals. The screening of a curated list of 198 epilepsy-related genes did not reveal any other pathogenic variants in this family. Total coverage was calculated for both whole exomes. The percentage of exonic bases with more than 10 × coverage was 69.4% in the proband and 84.7% in the mother.

## Discussion

Mutations in *SLC20A2* account for the majority of cases with IBGC [11, 19]. This condition is characterized by phenotypic variability spanning from lack of symptoms to a variety of progressive psychiatric and neurological symptoms [19].

Epilepsy has been reported only in seven such cases [1, 8, 11, 14, 25, 30], but only two reports focused on the epilepsy phenotype [8, 14]. Fjaer et al. reported two siblings with GTCSs easily controlled on antiepileptic medications. Both the patients also had a second pathogenic variant, in *CHRNB2*, a known epilepsy gene that might have contributed to the phenotype [8]. Knowles et al. reported refractory focal epilepsy in a pediatric patient who also had another variant, of unknown significance, in *SCN2A.* Thus the *SLC20A2* mutation contribution to the phenotype in this case remains uncertain. This individual also presented with dystonic events affecting the same hand that was also affected by seizures; the authors suggested that an accurate neurophysiology assessment was essential to classify epilepsy and differentiate it from the motor disorder [14].

A progressive myoclonus phenotype has been reported by Lamquet et al. in a patient bearing a likely pathogenic variant in *SLC20A2* [15]. However the authors did not specify whether the myoclonus was cortical, subcortical or spinal in origin. Their investigations (EEG and EMG) were uninformative and polygraphy was not performed.

Here we describe a two generations family bearing a newly-reported *SLC20A2* mutation. The screening of other candidate epilepsy genes did not reveal other pathogenic variants in this family.

The genetic analysis could only be performed for the proband and her mother. Her mother declined to undergo brain CT scan and did not volunteer any clinical symptoms, thus falling into the category of putatively incomplete penetrance [11, 19]. The proband’s two brothers declined genetic analysis; however, they did present with very similar clinical symptoms. They both underwent brain MRI that failed to show calcium deposition. This does not exclude the IBGC diagnosis as the exam was performed at the age of 18 and 22, respectively, while patients with this condition can first show basal ganglia calcification as late as at 40–50 years old [11, 18]. The brother (II:2) who had neurophysiological evaluation had findings consistent with the presence of cortical myoclonus.

The clinical presentation in this family is complex and includes both psychiatric and neurological conditions. We have focused on the neurological presentation as the presence of dystonia, cortical myoclonus and epilepsy in the proband proved challenging for both diagnosis and treatment strategies. To the best of our knowledge, cortical myoclonus has not been reported in IBGC before, possibly due to association with a new mutation in this pedigree, but possibly also because other cases may have been misdiagnosed with tremor considered part of a parkinsonian presentation. In our proband and in one of her brothers who agreed to testing, the EEG-EMG and the SEP analysis disclosed a myoclonus of cortical origin. The condition familial adult myoclonic epilepsy (FAME) is an autosomal dominant syndrome described in more than 70 families to date. Beyond myoclonus and seizures, psychiatric comorbidities seem to occur in this condition and a slow progression has also been described [3, 4]. Five different loci have been disclosed including one on chromosome 8 (8q24) [23] in Japanese families. The exact genetic defect reported for the Italian, Indian, French and Japanese families is similar and affects the non-coding DNA [5, 9, 12, 29]. For the Japanese families an expansion of an intronic ATTTT pentamer coupled with an insertion of a repeated ATTTC pentamer within the genes *SAMD12*, *TNRC6A*, *RAPGEF2* has been identified [12]. *SLC20A2* is located on 8p11.21, and basal ganglia calcification has never been reported in families with FAME [22]. Thus we do not consider our family has FAME.

Whilst the progressive nature may be reminiscent of some progressive myoclonic epilepsies (PMEs), these are usually associated with autosomal recessive inheritance. Franceschetti et al. reported the phenotypes of patients with PME of undetermined causes, showing they varied widely [10], but none included dystonia or brain calcification as seen in our kindred.

In our cases, seizures were an early symptom in all siblings, with an onset in childhood; seizures were not reported in their mother. Seizures were generalized, myoclonic or tonic–clonic, usually responded to VPA or LEV and worsened under CBZ. The proband presented with other events in adulthood, shown to be not epileptic. Some of them were clinically dystonic attacks, while some others were psychogenic. Myoclonus, on the contrary, was resistant to AED medication or only partially controlled at least in the proband.

The mutation found in our proband affects *SLC20A2*, a gene that account for the majority of cases with IBGC. This gene encodes for the inorganic phosphate (Pi) transporter PiT2, which is expressed in the whole brain, and mainly in the sites where calcifications tends to occur mostly, namely the globus pallidus, thalamus and cerebellum [6]. In the mouse brain PiT-2 has been detected in astrocytes, endothelial cells and neurons. *SLc20a2* Knockout mice show elevated CSF [Pi] suggesting that PiT2 is essential to export Pi from the CSF and thus to regulate the [Pi] in cerebrospinal fluid (CSF) [13]. Although we have not demonstrated directly the involvement of this transporter into the pathophysiology of myoclonus and epilepsy, we can hypothesize that the local cortical environmental ion balance might be altered and responsible for the seizures and myoclonus, or that the effect is indirect, following calcification. Indeed, excess phosphate is detrimental to glycogen structure, and in Lafora disease, there is over-accumulation of covalent phosphate in glycogen leading to a severe progressive myoclonus [26]. Moreover, myoclonus has been reported in basal ganglia calcifications of various other causes (for instance associated with mitochondrial encephalomyopathies, FORL1 deficiency) [21, 27].

Future studies will help explain why calcification is limited to specific parts of the brain, and the functional impact of the disordered phosphate homeostasis in the brain”.

In conclusion, we report one individual with *SLC20A2* mutation and a possibly associated new clinical presentation including cortical myoclonus. The variety of neurological symptoms and the overlapping psychiatric manifestation can prove challenging for diagnoses and management. The potential co-occurrence of different paroxysmal events, including epilepsy, dystonia, tremor and cortical myoclonus requires meticulous neurophysiology assessment to elucidate the origin of the events and to offer the best management.
